# A Bayesian Network Analysis of the Diagnostic Process and its Accuracy to Determine How Clinicians Estimate Cardiac Function in Critically Ill Patients: Prospective Observational Cohort Study

**DOI:** 10.2196/15358

**Published:** 2019-10-30

**Authors:** Thomas Kaufmann, José Castela Forte, Bart Hiemstra, Marco A Wiering, Marco Grzegorczyk, Anne H Epema, Iwan C C van der Horst, SICS Study Group

**Affiliations:** 1 Department of Anesthesiology University Medical Center Groningen University of Groningen Groningen Netherlands; 2 Department of Critical Care University Medical Center Groningen University of Groningen Groningen Netherlands; 3 Department of Clinical Pharmacology University Medical Center Groningen University of Groningen Groningen Netherlands; 4 Bernoulli Institute for Mathematics, Computer Science and Artificial Intelligence University of Groningen Groningen Netherlands; 5 Department of Intensive Care Maastricht University Medical Center+ Maastricht University Maastricht Netherlands

**Keywords:** cardiac function, physical examination, Bayesian network, critical care, ICU, medical education, educated guess, cognition, clinical decision-support, cardiology

## Abstract

**Background:**

Hemodynamic assessment of critically ill patients is a challenging endeavor, and advanced monitoring techniques are often required to guide treatment choices. Given the technical complexity and occasional unavailability of these techniques, estimation of cardiac function based on clinical examination is valuable for critical care physicians to diagnose circulatory shock. Yet, the lack of knowledge on how to best conduct and teach the clinical examination to estimate cardiac function has reduced its accuracy to almost that of “flipping a coin.”

**Objective:**

The aim of this study was to investigate the decision-making process underlying estimates of cardiac function of patients acutely admitted to the intensive care unit (ICU) based on current standardized clinical examination using Bayesian methods.

**Methods:**

Patient data were collected as part of the Simple Intensive Care Studies-I (SICS-I) prospective cohort study. All adult patients consecutively admitted to the ICU with an expected stay longer than 24 hours were included, for whom clinical examination was conducted and cardiac function was estimated. Using these data, first, the probabilistic dependencies between the examiners’ estimates and the set of clinically measured variables upon which these rely were analyzed using a Bayesian network. Second, the accuracy of cardiac function estimates was assessed by comparison to the cardiac index values measured by critical care ultrasonography.

**Results:**

A total of 1075 patients were included, of which 783 patients had validated cardiac index measurements. A Bayesian network analysis identified two clinical variables upon which cardiac function estimate is conditionally dependent, namely, noradrenaline administration and presence of delayed capillary refill time or mottling. When the patient received noradrenaline, the probability of cardiac function being estimated as reasonable or good P(E_R,G_) was lower, irrespective of whether the patient was mechanically ventilated (P[E_R,G_|ventilation, noradrenaline]=0.63, P[E_R,G_|ventilation, no noradrenaline]=0.91, P[E_R,G_|no ventilation, noradrenaline]=0.67, P[E_R,G_|no ventilation, no noradrenaline]=0.93). The same trend was found for capillary refill time or mottling. Sensitivity of estimating a low cardiac index was 26% and 39% and specificity was 83% and 74% for students and physicians, respectively. Positive and negative likelihood ratios were 1.53 (95% CI 1.19-1.97) and 0.87 (95% CI 0.80-0.95), respectively, overall.

**Conclusions:**

The conditional dependencies between clinical variables and the cardiac function estimates resulted in a network consistent with known physiological relations. Conditional probability queries allow for multiple clinical scenarios to be recreated, which provide insight into the possible thought process underlying the examiners’ cardiac function estimates. This information can help develop interactive digital training tools for students and physicians and contribute toward the goal of further improving the diagnostic accuracy of clinical examination in ICU patients.

**Trial Registration:**

ClinicalTrials.gov NCT02912624; https://clinicaltrials.gov/ct2/show/NCT02912624

## Introduction

### Background

In hemodynamically unstable patients admitted to the intensive care unit (ICU) for circulatory shock, the diagnosis and treatment decisions initially rely on accurate assessment of clinical examination [1,2]. Shock is the clinical expression of circulatory failure that results in inadequate cellular oxygen utilization and is often accompanied by systemic arterial hypotension, clinical signs of tissue hypoperfusion, and hyperlactatemia [3]. About one-third of critically ill patients experience circulatory shock, which is associated with increased morbidity and mortality [4].

Hemodynamic assessment of critically ill patients is challenging; depending on the type of shock, patients present with highly variable states of circulating blood volume, cardiac contractility, sympathetic nervous activity, vascular tone, and microcirculatory dysfunction. In addition, assessment is even more difficult if comorbidities are present [5]. Currently, hemodynamic estimates based on clinical examination show poor association with cardiac index in both univariate and multivariate analyses, and these estimates are no better than flipping a coin [6]. Due to this limited ability to assess a patient’s hemodynamic status using clinical examination, physicians often base changes in treatment primarily on information obtained through advanced monitoring techniques [7]. However, advanced monitoring techniques are currently advised and desired when clinical examination does not lead to a clear diagnosis, or when a patient does not respond to initial therapy [2,8]. Therefore, it is important to place emphasis on improving hemodynamic estimates made with clinical examination, to avoid inappropriate overuse of technological aid [9].

The first step in developing improved clinical examination structures for hemodynamic estimates is to study the current clinical practice. To understand how students and physicians diagnosed low cardiac index, Bayesian networks can be used to gain insight into the thought process behind the educated guess on hemodynamic status.

Bayesian networks have been frequently used to model domain knowledge in the context of decision support in other fields of medicine, given their ability to be interpreted as causal networks when no confounders are present [10-13]. By combining prior knowledge and the uncertainty in data, Bayesian networks allow for inference tasks to be performed, which establish conditional, possibly causal, dependencies between variables [14]. Conditional probabilities queries are interesting tools to study clinical reasoning, which are seen as an additive thought process where, at every step, information is interpreted conditioned on previously acquired information.

### Objectives

The aim of this study was to use Bayesian networks to investigate the decision-making process underlying estimates of cardiac function of patients acutely admitted to the ICU, based on current standardized clinical examination using Bayesian methods. Additionally, we aimed to determine the diagnostic accuracy of the current standardized clinical examination for estimating cardiac function in patients acutely admitted to the ICU.

## Methods

### Design, Setting, and Participants

This study was a predefined substudy of the prospective observational cohort Simple Intensive Care Studies-I (SICS-I) (ClinicalTrial.gov trial registration: NCT02912624) [15]. The study was approved by the local institutional review board (METc M15.168207). In SICS-I, all consecutive, acutely admitted adults expected to stay beyond 24 hours were included on their first day of admission to the ICU. Written informed consent was obtained from all patients or their relatives. This study is reported following the Standards for the Reporting of Diagnostic Accuracy Studies guidelines [16].

### Aims

The primary aim was to determine the conditional probabilities relating the variables measured during clinical examination to the cardiac function estimate made by the examiners.

The secondary aim of this study was to assess the diagnostic accuracy of cardiac function estimates made by the examiners and compare them to the cardiac index measured by critical care ultrasonography (CCUS).

### Bayesian Network Analysis

Bayesian networks are probabilistic models that represent the conditional (in)dependence relations between a set of variables in the form of a directed acyclic graph. In the graph, each variable is represented as a node and the directed edges (arcs) connecting the nodes represent the conditional dependency relations among the variables. Given the conditional (in)dependencies implied by the directed acyclic graph, the joint probability distribution of all variables can be factorized into a product of simpler local probability distributions.

From the initial set of variables registered during clinical examination, 14 clinical variables available from bedside monitors and patient record files, perfusors, physical examination, and the cardiac function estimate were included for modeling (Multimedia Appendix 1). All continuous variables were discretized according to the definitions provided in the study protocol. The correlation coefficients between variables after discretization were calculated with the Cramér V test for correlation strength.

The network structure was learned using the Max-Min Hill-Climbing algorithm with the Bayesian-Dirichlet equivalent scoring metric, as implemented in the R package “bnlearn” [17]. The Max-Min Hill-Climbing algorithm searches for the best network structure (ie, the best directed acyclic graph) that maximizes the Bayesian-Dirichlet equivalent scoring metric. To this end, the algorithm starts with an initial directed acyclic graph and then improves the Bayesian-Dirichlet equivalent score by iteratively adding, deleting, and reversing individual edges until the Bayesian-Dirichlet equivalent score does not improve further [18].

A set of restrictions can be applied to enforce certain connections between arcs in the network, so that prior knowledge is implemented *a priori* [13]. Arcs representing known dependencies can be whitelisted (ie, forced to appear in the directed acyclic graph), while arcs that represent impossible dependencies can be blacklisted (ie, excluded from the directed acyclic graph). In this network, *age* and *gender* are not determined by any other variables, so all arcs from other variables to these two were blacklisted. Similarly, as *estimate* does not influence any clinical variable, any arc from *estimate* to other variables was also blacklisted.

After the restrictions are defined, to obtain a confidence measure for the presence and directionality of the individual network edges, the bootstrap technique was applied. R=2000 bootstrap samples were generated from the original data, and the Max-Min Hill-Climbing algorithm was used to search for the best network for each bootstrap data set. This gives R=2000 best networks, and the confidence on the presence of an edge ranges from 0 (learned from 0 bootstrap samples) to 1 (learned from all bootstrap samples) [13]. To further increase the robustness of the final or consensus network, we defined the minimum significance threshold for arc strength as 0.700 if the calculated significance threshold was lower and accepted the calculated threshold otherwise. Regarding directionality, arcs with a direction coefficient below 0.666 after bootstrapping were considered undirected.

To determine the distributions of the variables and calculate the associated probabilities of the network, the adjacency matrix of the average bootstrapped directed acyclic graph was reproduced using the Bayesian network function, and belief propagation was carried out using the *gRain* package [13,19]. Belief propagation allows for inference tasks (probability queries) to be performed on the learned Bayesian networks, thereby providing a calculation of the distribution of values of a certain variable and the marginal and conditional probabilities of these values occurring based on the known value of an observed variable. Given a certain distribution, the marginal probability of a certain value occurring is calculated by integrating out all other variables, while the conditional probability is the probability of a value occurring for one variable, given a known, fixed value for at least one other variable [20]. These probability queries will allow for multiple relevant clinical scenarios to be recreated, based on the consensus network and the properties of the Markov blanket. When carrying out a query for *estimate*, if the values of its parent nodes are known, no other node can influence the conditional distribution of *estimate* [21]. If only some of its parent nodes are known, however, then some of the ancestors upstream of the undefined parent nodes can still influence the conditional probability of *estimate* [21]. To validate the structure learning process beyond the bootstrapping strategy used in learning a consensus network, two steps were taken. First, an ad hoc expert analysis was conducted to assess the plausibility and accuracy of the physiological relationships identified in the network. Second, 10-fold cross-validation was used to determine its predictive accuracy. Using the consensus network, the accuracy of the cross-validated predictions was determined by dichotomizing the estimates as described below and by calculating the area under the receiver operating curve, specificity, and sensitivity of the predictions made for patients, from which a validated cardiac index measurement was available.

### Definitions and Bias

Patients underwent a protocolized and standardized clinical examination and subsequent CCUS, as described in the SICS-I protocol [15]. The main variable of interest was cardiac function estimation made by the student or physician after clinical examination was performed but before CCUS was performed. Examiners could score cardiac function as “poor,” “moderate,” “reasonable,” or “good.” For diagnostic test analyses and the validation step of the network structure, the “poor” and “moderate” estimates were grouped as “low,” and the “reasonable” or “good” estimates were grouped as “high.” Quality of the CCUS images and measurements of cardiac index were validated by core laboratory technicians (Groningen Image Core Lab, Groningen, The Netherlands) who were blinded for the rest of the measurements. Cardiac index measurements were categorized in two groups: “low” for cardiac index≤2.2 L/min/m^2^ and “high” for cardiac index>2.2 L/min/m^2^ [22]. All patients for whom a validated cardiac index measurement and estimate of cardiac function were available were included in the Bayesian network analysis. Patients for whom CCUS images were of insufficient quality or cardiac index measurements were not available, were excluded from the diagnostic accuracy analysis.

### Statistical Analysis

Due to the observational nature of the study, a formal sample size calculation was not possible. Statistical analyses were performed in STATA 15.0 (StataCorp, College Station, Texas) and R version 3.5.1 (R Core Team, Vienna, Austria). Data are presented as mean with SD when normally distributed, or as median with interquartile range in case of skewed data. Dichotomous and categorical data are presented in proportions. Sensitivity and specificity for both the network’s and the examiners’ estimated guess were calculated by cross-tabulation of the respective predictions and the validated cardiac index measurements. Additionally, positive predictive values (PPV) and negative predictive values (NPV) and positive likelihood ratios (LR+) and negative likelihood ratios (LR-) were calculated with 95% CIs for the examiners’ estimates. For these, the overall accuracy was further expressed as a proportion of correctly classified cardiac index measurements (true negative and true positive measures) among all measures.

## Results

### Participants

A total of 1075 patients fulfilled our inclusion criteria, of which 1073 patients had available cardiac function estimates and were therefore included in the Bayesian network analysis. Of the included patients, 783 (73%) had validated cardiac index measurements and were included in the diagnostic accuracy tests. Further, 569 patients (73%) were included by students and 214 patients (27%) were included by physicians.

### Descriptive Measures

Characteristics of included patients according to availability of cardiac index measurements are shown in Table 1. Body mass index and Simplified Acute Physiology Score (SAPS) II score were significantly different between patients (Table 1).

### Bayesian Network Analysis

The structure learned for the network identified two clinical variables, namely, noradrenaline administration and the presence of delayed capillary refill time or mottling (dCRT-M), upon which the estimates of cardiac function are directly conditionally dependent (Table 2).

**Figure 1:**
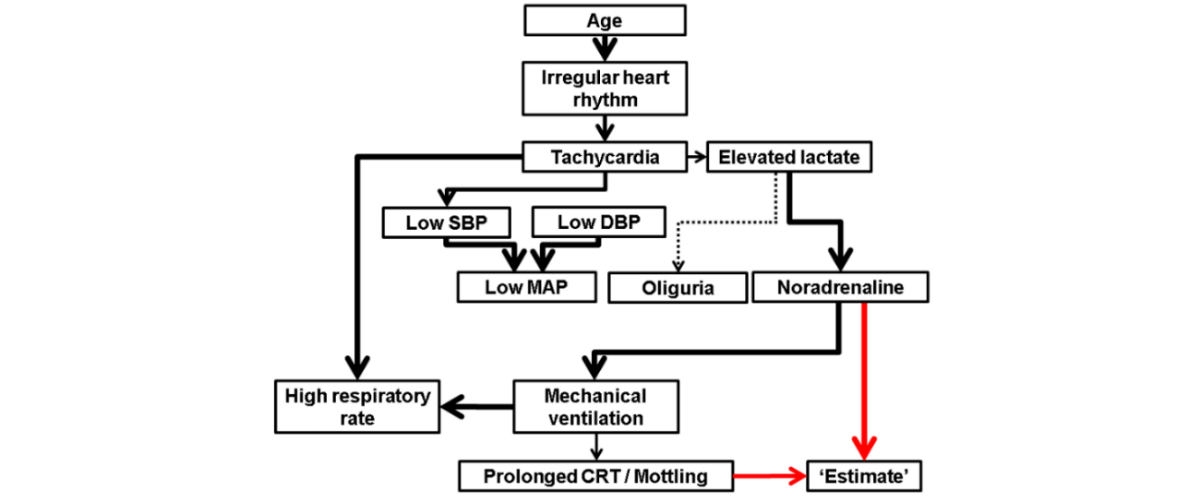
Consensus directed acyclic graph. Red lines represent direct conditional dependencies to estimate. Black lines represent direct conditional dependencies to other variables. Width of the line represents strength coefficient. The dotted line represents the weakest strength coefficient. DBP: diastolic blood pressure; SBP: systolic blood pressure; MAP: mean arterial pressure; CRT: capillary refill time.

As denoted in Figure 1 by the dotted line, the arc from elevated lactate to oliguria had the lowest strength coefficient (0.728). The average directionality coefficient was 0.909, indicating well-defined directionality. Only one edge (between mechanical ventilation and high respiratory rate) did not meet the threshold for directionality and was thereby left undirected in the consensus directed acyclic graph (for querying, however, a direction from high respiratory rate to mechanical ventilation was defined based on expert knowledge to comply with the formal computational requirements) [15]. Additionally, there was no difference in network structure when including only students (n=801) or only physicians (n=271) compared to the network obtained with all the participants’ estimates.

**Figure 2:**
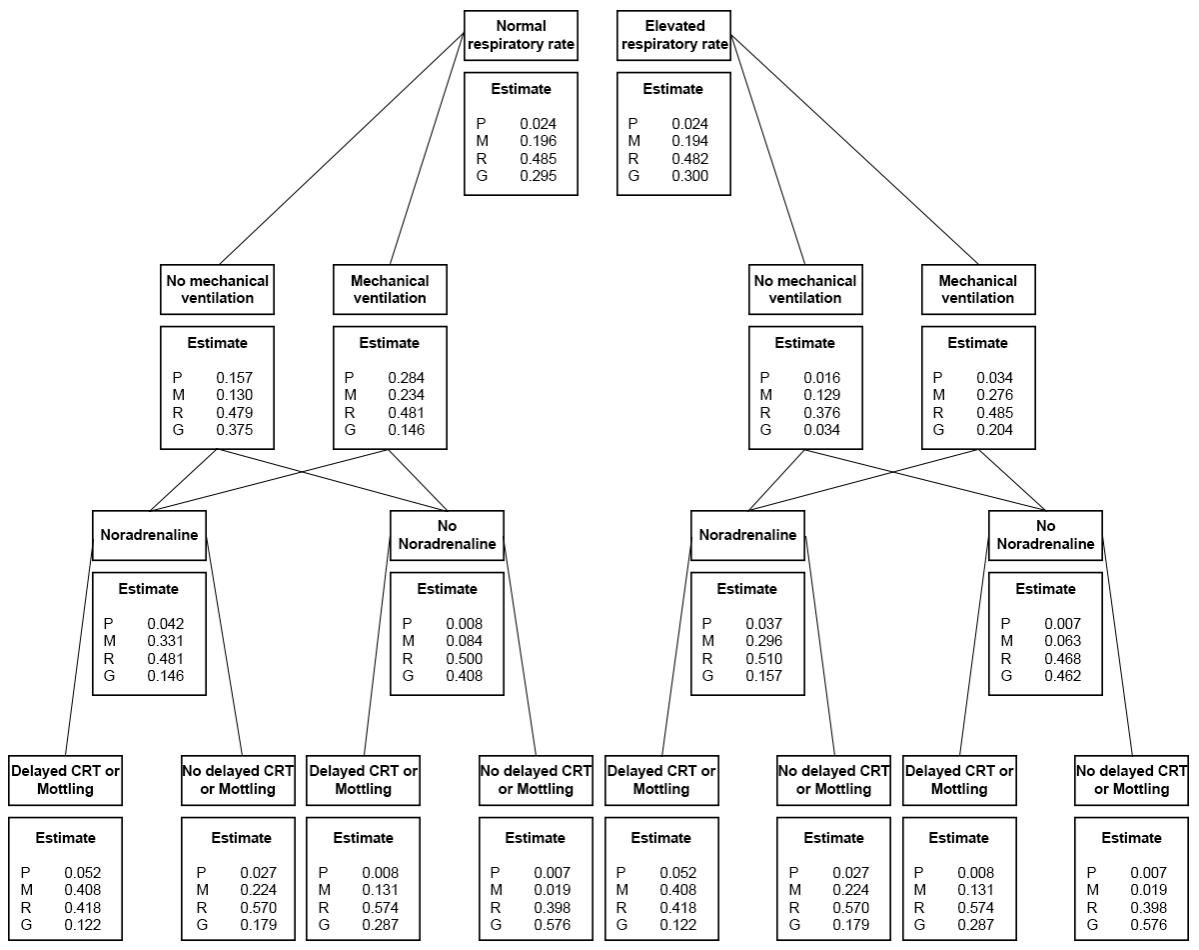
Tree diagram showing the conditional probabilities queries for estimate associated with multiple scenarios during clinical examination. At each step, only the variables above the split are known and as more information becomes available, the conditional probabilities change. P=Poor; M=Moderate; R=Reasonable; G=Good; CRT: capillary refill time.

The probability queries conducted with the conditional probabilities for *estimate* are presented in a tree diagram in Figure 2. Each of the pathways in the diagram represents a scenario that could occur during clinical examination. Since one of the main focuses of SICS-I was the collection and interpretation of information available at bedside during physical examination, we expanded the conditional probability queries to also include respiratory rate and mechanical ventilation. Tachypnea virtually did not influence the probability of cardiac pump function being estimated as reasonable or good P(E_R,G_), whereas ventilation status did (P[E_R,G_|not ventilated, no tachypnea]=P[E_R,G_|not ventilated, tachypnea)=0.85; P[E_R,G_|ventilated, tachypnea]=0.69 and P[E_R,G_|ventilated, no tachypnea]=0.63). When the patient received noradrenaline, P(E_R,G_) was lower irrespective of whether they were mechanically ventilated (P[E_R,G_|ventilation, noradrenaline]=0.63, P[E_R,G_|ventilation, no noradrenaline]=0.91, P[E_R,G_|no ventilation, noradrenaline]=0.67, P[E_R,G_|no ventilation, no noradrenaline]=0.93). The same trend was found for dCRT-M, with reasonable or good estimates being more likely in the absence of dCRT-M.

Finally, an area under the receiver operating characteristic curve of 0.58 was obtained for the 10-fold cross-validated predictions of cardiac function made by the consensus network, with a specificity of 36% and a sensitivity of 79% [23].

**Table 1:** Patient characteristics.

Variable		No cardiac index measurement (n=292)	Cardiac index measurement (n=783)	Total (N=1075)	*P* value
Age (years), mean (SD)		62 (14)	62 (15)	62 (15)	.75
Male gender, n (%)		188 (64)	486 (62)	674 (63)	.49
Body mass index (kg/m^2^), mean (SD)		27.5 (5.4)	26.7 (5.6)	26.9 (5.5)	.04
Arterial pressure (mm Hg), mean (SD)		78 (14)	79 (14)	78 (14)	.30
Heart rate (bpm^a^), mean (SD)		87 (22)	88 (21)	88 (21)	.35
Irregular heart rhythm, n (%)		28 (10)	88 (11)	116 (11)	.44
Central venous pressure (mm Hg), median (IQR)		9 (5, 12)	9 (5, 13)	9 (5, 13)	.74
Patients administered noradrenaline, n (%)		142 (49)	386 (49)	528 (49)	.85
Urine output (mL/kg/h), median (IQR)		0.6 (0.3, 1.2)	0.7 (0.4, 1.2)	0.6 (0.4, 1.2)	.22
Respiratory rate (bpm), mean (SD)		18 (5)	18 (6)	18 (6)	.50
Mechanical ventilation, n (%)		179 (61)	452 (58)	631 (59)	.29
Positive end-expiratory pressure (cm H_2_O), median (IQR)		7 (5, 8)	7 (5, 8)	7 (5, 8)	.41
Central temperature (°C), mean (SD)		37.0 (0.9)	36.9 (0.9)	36.9 (0.9)	.84
Difference between central temperature and temperature of the dorsum of the foot (°C), mean (SD)		7.7 (3.2)	7.8 (3.2)	7.8 (3.2)	.66
Subjective “cold” temperature, n (%)		109 (37.6)	289 (37.1)	398 (37.2)	.88
**Capillary refill time**					
	Knee (s), median (IQR)	3.0 (2.0, 4.5)	3.0 (2.0, 4.5)	3.0 (2.0, 4.5)	.48
	Sternum (s), median (IQR)	2.8 (2.0, 3.0)	3.0 (2.0, 3.0)	3.0 (2.0, 3.0)	.84
	Finger (s), median (IQR)	3.0 (2.0, 4.0)	2.5 (2.0, 4.0)	2.5 (2.0, 4.0)	.37
**Mottling rate, mean (SD)**					.64
	None	157 (58.8)	397 (56.8)	554 (57.3)	
	Mild	24 (9.0)	79 (11.3)	103 (10.7)	
	Moderate	75 (28.1)	201 (28.8)	276 (28.6)	
	Severe	11 (4.1)	22 (3.1)	33 (3.4)	
Hemoglobin (mmol/L), mean (SD)		6.8 (1.5)	6.8 (1.4)	6.8 (1.4)	.90
Lactate (mmol/L)		1.4 (0.9, 2.4)	1.4 (0.9, 2.2)	1.4 (0.9, 2.2)	.79
ICU^b^ length of stay (days)		3.5 (1.9, 6.9)	3.1 (1.9, 6.5)	3.2 (1.9, 6.6)	.29
SAPS^c^ II (points)		47 (37, 58)	44 (34, 56)	45 (35, 57)	.037
APACHE^d^ IV score (points)		77 (56, 92)	73 (55, 91)	74 (56, 92)	.14
90-day mortality, n (%)		81 (27.7)	217 (27.7)	298 (27.7)	.99
**Cardiac function estimate, n (%)**					.004
	Poor	8 (2.8)	18 (2.3)	26 (2.4)	
	Moderate	46 (15.9)	165 (21.1)	211 (19.7)	
	Reasonable	164 (56.6)	349 (44.6)	513 (47.8)	
	Good	72 (24.8)	251 (32.1)	323 (30.1)	

^a^bpm: beats per minute.

^b^ICU: intensive care unit.

^c^SAPS: Simplified Acute Physiology Score.

^d^APACHE: Acute Physiology and Chronic Health Evaluation.

**Table 2:** Strength and direction coefficients of the consensus directed acyclic graph.

From	To	Strength	Direction
Age	Irregular rhythm	0.983	1.00
Mechanically ventilated	High respiratory rate	0.994	0.504
Mechanically ventilated	dCRT-M^a^	0.875	0.884
Irregular rhythm	Tachycardia	0.848	0.954
Tachycardia	High respiratory rate	0.999	0.931
Tachycardia	Low SBP^b^	0.821	0.883
Tachycardia	Elevated lactate	0.832	0.821
Low SBP	Low MAP^c^	1	1
Low DBP^d^	Low MAP	1	1
Elevated lactate level	Oliguria	0.728	0.803
Elevated lactate level	Noradrenaline administration	1	1
Noradrenaline administration	Mechanically ventilated	1	0.957
Noradrenaline administration	Estimate	0.999	1
dCRT-M	Estimate	0.876	1

^a^dCRT-M: delayed capillary refill time or mottling.

^b^SBP: systolic blood pressure.

^c^MAP: mean arterial pressure.

^d^DBP: diastolic blood pressure.

### Diagnostic Accuracy

Diagnostic accuracy tests for estimating of a low cardiac index showed a sensitivity of 26% and 39%, a specificity of 83% and 74%, PPV of 45% and 48%, NPV of 67% and 66%, LR+ of 1.52 and 1.52, and LR- of 0.89 and 0.82 for students and physicians, respectively. The overall accuracy of cardiac index estimates was 63% and 61% for students and physicians, respectively. For all patients combined, sensitivity was 30%, specificity was 80%, PPV was 46%, NPV was 67%, LR+ was 1.53, LR- was 0.87, and the overall accuracy of diagnostic tests was 62% (Table 3).

**Table 3:** Accuracy, sensitivity, specificity, predictive values, and likelihood ratios for students’ and physicians’ estimates.

Variable	Students (n=569)	Physicians (n=214)	Overall (N=783)
Sensitivity, % (95% CI)	26 (20-33)	39 (28-50)	30 (25-36)
Specificity, % (95% CI)	83 (78-86)	74 (66-82)	80 (77-84)
Positive predictive value, % (95% CI)	45 (38-53)	48 (39-58)	46 (40-53)
Negative predictive value, % (95% CI)	67 (65-69)	66 (61-71)	67 (65-69)
Positive likelihood ratio, 95% CI	1.52 (1.10-2.09)	1.52 (1.02-2.25)	1.53 (1.19-1.97)
Negative likelihood ratio, 95% CI	0.89 (0.81-0.98)	0.82 (0.67-1.00)	0.87 (0.80-0.95)
Overall accuracy, % (95% CI)	63 (59-67)	61 (54-67)	62 (59-66)

## Discussion

### Principal Findings

Clinical examination is used daily by physicians as an easy, cheap, and noninvasive way of gathering information to guide interventions and further diagnostic testing. Clinical signs such as oliguria; altered consciousness; and cold, clammy skin are known possible indicators of organ hypoperfusion and are used to diagnose shock in critically ill patients [2]. However, the value of clinical examination has been questioned, and previous studies have shown physicians to perform poorly in diagnosing a low cardiac index based on physical signs alone [8,9]. In this study, we confirmed that the accuracy of these estimates remains low for both students and physicians. Surprisingly, we identified noradrenaline administration and delayed CRT or mottling as seemingly the major factors influencing cardiac function estimates using Bayesian network analysis. These findings may serve as the basis for improving the value of clinical examination (1) by identifying some of the biases clinicians may be subjected to, which causes them to overdiagnose compared to students, and (2) by clarifying some of the thought process behind the clinical examination. This allows the examiner to “think about how they think” when performing clinical examination and can help clinicians be trained to prioritize or leave out certain variables when making their assessment.

### Bayesian Network Analysis

#### Validation and Limitations

Validation of the network structure was a crucial yet challenging step toward our goal of trying to obtain a plausible representation of the examiners’ knowledge network and thought process at bedside. We believe to have tackled this challenge in the best way possible by validating it in three different ways: using the bootstrapping process to generate a consensus network; conducting expert validation of the plausibility of the arcs; and using the network as a predictor, as previously suggested [13]. We believe that the similarity in accuracy, sensitivity, and specificity between the network’s predictions and the examiners’ own estimates is further proof of the validity of its structure. It must be restated that the goal of this study was not to build and optimize a predictive model, in which case the predictive accuracy, sensitivity, and specificity we obtained would be subpar. In fact, had the network been able to make the estimates with a substantially higher accuracy than the examiners’ estimates, we would be more reluctant to affirm that is parallel with the examiner’s thought process.

As any exploratory study, however, we faced several limitations. The first was practical, as not all included patients had cardiac index measurements, since CCUS is not applicable for every ICU patient and views obtained by CCUS can be obstructed due to lines, wounds, or excess adiposity [24]. This prevented us from using the complete cohort and likely accounted for the difference in SAPS-II score and body mass index in the patients with and without CCUS measurements. Second, the discretization required by the parametric assumptions of Bayesian network algorithms comes with the inherent risk of useful information being discarded in the process, which does not guarantee that the dependence relationships involving the original variables are preserved. Last, for causality to be derived from Bayesian networks, there must be no unobserved variables influencing the variables included in the network that may act as confounding factors. In SICS-I, the focus was on examining and improving students’ and physicians’ educated guess, resorting primarily to bedside information, such as vasopressor and fluid perfusors, vital signs, and physical examination. Therefore, to best replicate this scenario, we opted to include in the network only variables that are readily available during the protocolized examination. Although this increases the risk of introducing bias in the causal network, the accuracy of the physiological dependencies identified gives us reason to believe that no substantial bias is present.

#### Do Probability Queries Help Explain the Modest Diagnostic Accuracy?

Previous studies on the diagnostic accuracy of clinical examination have found the performance of experienced physicians and students to be comparable [6]. Expert physicians are more often affected by multiple cognitive biases, such as confirmatory bias and premature closure, compared to students, who remain more open to new hypotheses and persist in collecting data [25,26]. Interestingly, while the diagnostic accuracy for individual physicians can be as low as 62.5%, there is a visible increase as the number of physicians involved increases (up to 85.6% for groups of nine physicians) [27]. Our results are in line with the literature, and we additionally showed that physicians had a higher sensitivity but lower specificity than students (39% and 26%, and 74% and 83%, respectively). These differences in sensitivity and specificity represent a tendency of physicians to overdiagnose, which has previously been related to confirmatory bias and premature closure. Indeed, two other findings support the idea already given by the direct dependence of *estimate* solely on noradrenaline and dCRT-M that premature closure was a common phenomenon. First, in the probability queries, while machine ventilation does not directly influence the *estimate*, considerable changes in the probability of the *estimate* are still observable, depending on whether the patient is ventilated, before noradrenaline use and dCRT-M are known. This could be due to the fact that mechanical ventilation is almost inevitably the first variable to be noted when the examiner approaches bedside. Second, a comparison of the change in the probabilities of *estimate* based on varying clinical evidence with the likelihood ratios calculated in another SICS-I substudy shows that variables further upstream of estimate such as respiratory should be taken more into account [15]. For example, while the positive and negative likelihood ratios of a high respiratory rate are as suggestive as those of a delayed CRT, the query shows that the probability of being estimated to have low cardiac function was considerably lower in those without dCRT-M (0.25) than in those with dCRT-M (0.46) and the probability of a patient with tachypnea being estimated to have low or high cardiac function was virtually the same. This is despite tachypnea having a positive and negative likelihood ratio of 1.16 and 0.68, respectively.

### Conclusion and Future Implications

This study confirms that the accuracy of cardiac function estimates remains low for both students and physicians, and it identifies noradrenaline administration and delayed CRT or mottling as seemingly the major factors influencing these estimates. Although it will remain challenging to try to replicate the thought process of the examiner, not only methodologically, but also because different individuals have different levels of knowledge and different examination routines, Bayesian networks seem like a promising tool to help break down and better understand the educated guessing process. The insight gained in studies such as this one, can help teach students think about how they think and, on a clinical level, provide much-needed guidance for prioritization of variables during clinical examination. In fact, our team is currently compiling the knowledge acquired in the SICS-I substudies to build an interactive game for medical students, residents, and specialists. This electronic learning tool will ask the player to estimate cardiac function using the same scale and data from variables such as bedside monitor hemodynamic variables, ventilator and pump settings, and urine output.

## Appendix

Multimedia Appendix 1 Variables included in the Bayesian network and the respective Cramér V similarity measure.

## Abbreviations

APACHE: Acute Physiology and Chronic Health Evaluation
CCUS: critical care ultrasonography
DBP: diastolic blood pressure
dCRT-M: delayed capillary refill time or mottling
ICU: intensive care unit
LR-: negative likelihood ratios
LR+: positive likelihood ratios
MAP: mean arterial pressure
NPV: negative predictive values
PPV: positive predictive values
SAPS: Simplified Acute Physiology Score
SBP: systolic blood pressure
SICS-I: Simple Intensive Care Studies-I
