# Supplementary material for: A Bayesian Network Analysis of the Diagnostic Process and its Accuracy to Determine How Clinicians Estimate Cardiac Function in Critically Ill Patients: Prospective Observational Cohort Study
Source: JMIR Med Inform. 2019 Oct 30;7(4):e15358. doi: 10.2196/15358 (PMC6913745; doi:10.2196/15358)
Supplement: Multimedia Appendix 1 [file medinform_v7i4e15358_app1.pdf]

SUPPLEMENT 1: Variables included in the Bayesian network and the respective Cramér's V similarity measure.

Making an educated guess of cardiac function in critically ill patients using clinical examination: lessons from a Bayesian network analysis to improve diagnostic accuracy

Thomas Kaufmann[1]*, MD, Jose Castela Forte[1,2,3,4]*, BSc, Bart Hiemstra[2], MD PhD, Marco A Wiering[4], PhD, Marco A Grzegorczyk[4], PhD, Anne H Epema[1], MD PhD, Iwan CC van der Horst[2], MD PhD, SICS study group

* Both authors contributed equally to this manuscript

	Age	Gender	MVent	Irreg HR	Tachy	HRR	Low SBP	LowDBP	LowMAP	eLactate	Oliguria	Delta T	Nor	dCRT-M	Estimate
**Age**	1.00	0.03	0.03	0.27	0.08	0.01	0.06	0.07	0.05	0.08	0.14	0.13	0.08	0.12	0.19
**Gender**		1.00	0.04	0.04	0.02	0.02	0.00	0.01	0.05	0.04	0.02	0.04	0.02	0.02	0.10
**MVent**			1.00	0.04	0.00	0.17	0.07	0.06	0.04	0.09	0.00	0.11	0.35	0.18	0.19
**Irreg HR**				1.00	0.14	0.01	0.05	0.04	0.03	0.00	0.02	0.03	0.02	0.02	0.16
**Tachy**					1.00	0.23	0.17	0.00	0.09	0.19	0.10	0.00	0.12	0.04	0.08
**HRR**						1.00	0.04	0.00	0.06	0.13	0.02	0.06	0.02	0.02	0.12
**LowSBP**							1.00	0.04	0.46	0.16	0.10	0.05	0.22	0.07	0.29
**LowDBP**								1.00	0.55	0.05	0.08	0.00	0.11	0.03	0.16
**LowMAP**									1.00	0.15	0.09	0.02	0.20	0.03	0.29
**eLactate**										1.00	0.19	0.12	0.27	0.11	0.24
**Oliguria**											1.00	0.11	0.20	0.08	0.23
**Delta T**												1.00	0.20	0.17	0.23
**Nor**													1.00	0.14	0.42
**dCRT-M**														1.00	0.29
**Estimate**															1.00

Abbreviations: MVent, mechanically ventilated; Irreg HR, irregular heart rate; Tachy, tachycardia; HRR, high respiratory rate; SBP, systolic blood pressure; DBP, diastolic blood pressure; MAP, mean arterial

pressure; eLactate, elevated lactate; Delta T, large temperature variation; Nor, noradrenaline administration; dCRT-M, delayed capillary refill time and/or mottling.
